# Teleworking from home experiences during the COVID-19 pandemic among public health workers (TelEx COVID-19 study)

**DOI:** 10.1186/s12889-022-13031-0

**Published:** 2022-04-07

**Authors:** Josephine Sau Fan Chow, Dimetrious Palamidas, Sonia Marshall, Wendy Loomes, Suzie Snook, Rebecca Leon

**Affiliations:** 1 South Western Sydney Local Health District, 52 Scrivener St, Warwick Farm, Sydney, NSW 2170 Australia; 2 The University of Sydney, Sydney, Australia; 3 The University of New South Wales, Sydney, Australia; 4 Western Sydney University, Sydney, Australia; 5 University of Tasmania, Hobart, Australia

## Abstract

**Background:**

When working from home (WFH) became temporarily necessary for staff as a result of the COVID-19 pandemic in 2020, it had to be implemented without significant organisational experience or understanding of WFH and its complexities. This study aims to determine the impacts experienced by staff who have undertaken WFH during the COVID-19 pandemic.

**Methods:**

This was an observational cross-sectional study using survey with a purposive sampling strategy for staff from corporate and non-clinical departments. These staff undertook WFH during COVID-19 pandemic in 2020. None of these staff had any direct operational roles in a hospital facility and clinical service. Participants’ self-reports of their mood while working in their normal workplace and while WFH were collected via the Scale of Positive and Negative Experience (SPANE), a validated affect balance questionnaire. The responses from the open-ended question were analysed using thematic analysis approach.

**Results:**

A total of 143 participants completed the survey responses. Majority (61%) WFH for four or more months as a result of the COVID-19 pandemic. Participants rated their skills very highly on the technologies with an average rating of 9 (out of 10) for computer skills, smartphones and videoconferencing/teleconferencing applications. Participants felt WFH was an improvement on normal working, in particular in relation to their ability to concentrate and be productive. The “SPANE” relating to affect balance while WFH was completed by 124 participants (85.7%), resulting in a mean score of 5.45 (S.D. 2.98). The SPANE relating to normal working conditions was completed by 127 participant (88.8%) resulting in a mean score of 2.70 (S.D 3.69). This indicated that while participants’ positive emotions typically predominated in both situations, they felt slightly more positive on average with WFH. Over 90% participants reported that they would take the opportunity to WFH again if it were offered. Data obtained from the open-ended questions had complimented the findings of the structure close-ended questions in the benefits of remote working and support for their health and wellbeing. The open-ended questions had provided additional information on challenges which the participants encountered during the WFH experience and their suggested preference to sustain this workplace practice.

**Conclusions:**

This study highlighted factors that impacted workers’ work processes, productivity, physical and mental health well-being while WFH and provided a foundation for considering how to best support a positive WFH experience.

## Background

Teleworking is broadly defined as work that is performed remotely, away from a centralised workplace, using information and communication technologies (ICTs) to link with their employer [1]. Telework may take place in a variety of locations, such as satellite offices and co-working spaces, however it is frequently associated with working in the employee’s own home (working from home). Home-based telework is offered to employees of many organisations with the expectation that it will improve work-life balance and promote staff wellbeing. Some research support the benefits of home based telework, on its own, or as part of broader flexible working arrangements [2–4].

Reported benefits from working from home (WFH) included reducing work-family conflict and role overload [2]; lower worker stress levels, improved health and reduced absence from work [3, 5]; reduced depression levels in mothers of young children [4]; improved quality of life [6]. However such benefits had not been demonstrated consistently across different workforce and contexts, and in some cases WFH in general had been found to have negative consequences [7–10]. Studies report that WFH were not always necessarily beneficial to employees in terms of social, behavioural and physical factors [11–13]. A variety of contextual factors such as; the demands of the home environment, level of organisational support, and social connections external to work determined positive or negative impact of WFH on the worker [13, 14]. A recent study from California reported decreased overall physical and mental well-being of workers after WFH, associated with reduced physical activity, increased junk food intake, lack of communication with co-workers and children at home, distractions while working, adjusted work hours, workstation set-up and higher work load [15]. Evidence showed that inability to disengage home from work could increase stress and greater degree of self-reported exhaustion [10, 16]. Virtual teamwork necessitated by WFH lacked the communication richness of face to face interaction and had the potential to escalate workplace conflict. Research showed that it might impact workers’ creativity and opportunities to seek help and support, the management relationships, in particular on opportunities for feedback, learning & performance management [11]. Feeling stressed due to self-isolation, which in turn could affect their feelings about work [7].

The benefits of WFH might be greater for single people than those with partners and/or children. The existing literature showed disparities in the impact of WFH relating to gender, which included improved work-life-balance experienced by men engaged in WFH but not by women, and a tendency for men to use WFH to devote more time to work, while women used such flexibility to devote more time to home duties and child care [9, 12]..

Despite previous research identifying the option to WFH as important in facilitating worker compliance with pandemic control measures [17], prior to the COVID-19 pandemic, the option to WFH was not broadly available to most employees in public health system. While many health staff had experience of teleworking from across sites within the various sites and certain staff meeting the criteria for WFH under the Fair Work Act 2009 [18] might have been allowed to WFH, no policies exist outlining how WFH should be implemented and managed. Thus, when WFH became temporarily necessary for staff as a result of the COVID-19 pandemic in 2020, it had to be implemented without significant organisational experience or understanding of WFH and its complexities, leaving considerable room for unanticipated issues and consequences to arise from the sudden, mass transition to an unfamiliar way of working.

This study aims to determine the impacts experienced by staff who had undertaken telework from home during the COVID-19 pandemic in 2020.

## Methods

### Design

This was an observational cross-sectional study using survey with a purposive sampling strategy for staff from corporate and non-clinical departments.

### Participant and recruitment

The participants were employee who provided support roles and functions at one of the following departments, within a large local health district in Sydney, Australia: the Innovation and Business Unit, Nursing and Midwifery Administration, Research Directorate, Capital Works, Planning Unit, People & Culture, Information Communication and Technology Service, Finance and Clinical Governance Unit. These employee undertook WFH when it became temporarily necessary for staff as a result of the COVID-19 pandemic between March to July 2020. None of these employee had any direct operational roles in a hospital facility and clinical service.

Employee from the participating departments were invited via email and link to complete an anonymous online survey and provided with a message outlining the purpose and content of the survey, its voluntary and anonymous nature and the ways in which the data would be shared and reported. Survey questions included demographic information, normal working arrangements (e.g. full or part time) and previous experience of remote working. Questions relating to participants’ experiences of home-based telework during COVID-19 covered 3 key domains identified by previous research relating to the impacts of telework and flexible working arrangements. They were: work processes and productivity, family and home, and, personal wellbeing. The personal wellbeing section of the survey were adopted from the Scale of Positive and Negative Experience (SPANE) [20], a validated affect balance questionnaire, to record the self-reported frequency with which participants experience positive and negative emotions during WFH and normal working arrangements. Participants were asked to rate, using a Likert scale, how often they experiences a variety of positive and negative emotional states. The scoring of the SPANE results in an ‘affect balance’ score ranging from 24 to 24, with negative scores indicating predominantly negative affect and positive scores indicating predominantly positive affect. Participants were also asked to report their preferences in relation to working from home in the future, in order to gauge whether their experiences during COVID-19 pandemic had affected their preference. Close-ended questions were used to allow for ease of analysis and open-ended questions were also offered to capture any experiences or impacts which did not fit into the pre-defined domains.

### Data collection and analysis

Percentages were calculated for categorical variables, using the denominator of the number of valid responses for the data item. Mean and standard deviation were calculated for continuous variables. For questions with 10 point Likert scale, the mean and SD was calculated for each response on the scale of 1–10. The Pearson’s Chi-square test was used for analysis of categorical variables and the Wilxocin-Kruskal-Wallies test for the analysis of continuous variables. A *p*-value less than 0.05 was considered statistically significant. Analysis were undertaken using the SAS Software (Version 8.2). The responses from the open-ended questions and comments were analysed using thematic analysis approach. Ethics approval for this study was granted by the Human Research Ethics Committee.

## Results

### Participant demographics

A total of 160 survey (60.6% response rate) were received between 10 February 2021 to 31 May 2021. Seventeen responses were excluded from analysis as the participants only completed the demographic section of the survey or indicated via their responses that they never worked from home during the COVID-19 pandemic. More than 65% (*n* = 94) of the participants were female, 75.5% (*n* = 108) between 31–60 year old and with 79.7% (*n* = 114) completed a higher educational training (Table 1).

**Table 1:** Demographic profile of participants (*n* = 143)

Variables	N (%)
**Gender**	
Male	48 (33.6%)
Female	94 (65.7%)
No Response	1 (0.7%)
**Age in years**	
21–30	19 (13.3%)
31–40	39 (27.3%)
41–50	38 (26.5%)
51–60	31 (21.7%)
61–70	14 (9.8%)
No Response	2 (1.4%)
**Lives with a partner**	
Yes	107 (74.8%)
No	36 (25.2%)
**Number of other adults in household (other than a partner)**	
0	104 (72.7%)
1	13 (9.1%)
2	19 (13.2%)
3	5 (3.5%)
4	2 (1.4%)
**Number of children in the household**	
0	67 (46.8%)
1	20 (13.9%)
2	41 (28.7%)
3	5 (3.5%)
4	3 (2.1%)
No Response	7 (4.9%)
**Highest level of education**	
high school	11 (7.7%)
certificate	11 (7.7%)
diploma	11 (7.7%)
bachelor degree	38 (26.5%)
graduate certificate or diploma	8 (5.6%)
postgraduate degree	57 (39.9%)
No Response	7 (4.9%)
**Born in Australia**	
Yes	78 (54.5%)
No	64 (44.8%)
No Response	1 (0.7%)
**Spoke English as first language**	
Yes	94 (65.7%)
No	47 (32.9%)
No Response	2 (1.4%)
**Indigenous status**	
Aboriginal	1 (0.7%)
Torres Strait Islander	0
Both	1 (0.7%)
Neither	139 (97.2%)
No Response	2 (1.4%)
**Person with a disability**	
Yes	5 (3.5%)^a^
No	134(93.7%)
No Response	4 (2.8%)

^a^Participants were also asked whether their disability necessitated workplace adjustments, however none responded to this question

Fifty participants (34.7%) provided services directly to health consumers (patients, clients, care recipients, etc.). Fifty participants (34.7%) managed other staff. Ninety-two (63.8%) provided supporting role (e.g. finance, human resources) that did not interact directly with health consumers. Two participants did not respond. Participants worked an average of 68 h in a typical fortnight (SD = 18.3). 119 participants were permanent employees, 20 were temporary, 2 were causal and 2 did not provide their employment status. Participants were also asked to rate their skills in using and understanding various technologies on a scale from 1 (basic) to 10 (advanced). Overall, participants rated their skills very highly on all 3 technologies with an average rating of 9 for computer skills, smartphones and videoconferencing/teleconferencing applications.

### Previous experiences with telework

Seventy participants (48.9%) reported previous experience of WFH. Twenty-six (18.2%) of these participants felt that it had been easier to obtain approval to WFH prior to COVID-19 than it was during the pandemic. Seventy participants (48.9%) reported that they had no previous experience of WFH. Of these, 32 (22.4%) previously desired/requested the opportunity to WFH. Eighty-seven participants (60.8%) had experience working across multiple sites as part of their job, as opposed to WFH specifically.

### Telework arrangements during COVID-19

**Fig. 1:**
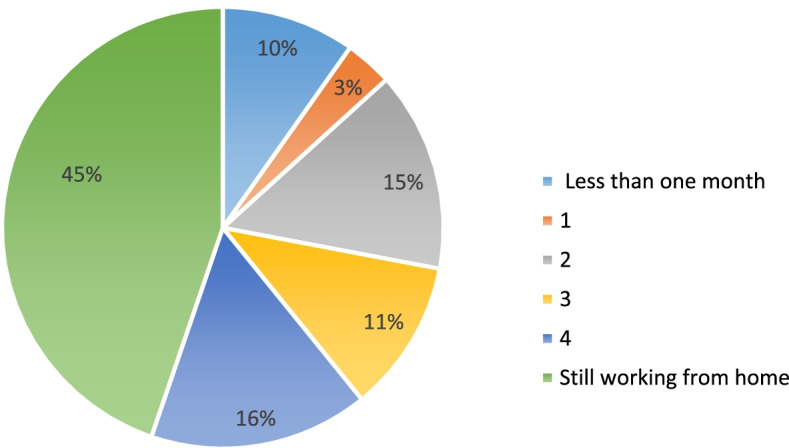
Duration of telework from home during COVID-19

Only 17 participants (11.9%) reported working exclusively at home during COVID-19 pandemic, while the remainder alternated between home and their usual workplace. Participants’ responses indicated that the majority (61%) WFH for four or more months during pandemic period. Fourteen participants (9.8%) reported their WFH arrangement ended after less than a month (Fig. 1).

**Fig. 2:**
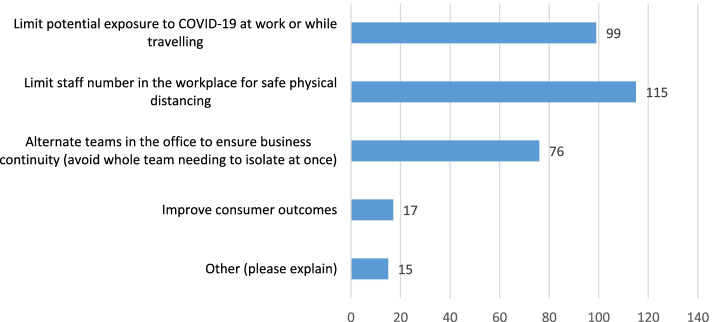
Reasons offered for telework arrangements during COVID-19

Majority of participants were offered justification for their WFH arrangement during COVID-19 pandemic with the main reasons to limit staff number in the workplace for safe physical distancing and also limit potential exposure to COVID-19 at work while travelling (Fig. 2).

Fifteen participants reported that WFH were offered to them so as to vacate their usual office space for pandemic-related activities. Other reasons cited included specific medical advice to WFH due to underlying health problems, the need to care for children at home and a pre-existing norm within the department that WFH was allowed. Seven participants (4.9%) reported being unable to access all their normal work resources while WFH. These were mainly technological resources such as printers, shared drives and their preference for desktop versus laptop computers. Forty-nine participants (34.3%) reported that adjustments had to be made to their home environment in order to make it suitable for WFH.

### Impact of teleworking on work processes and productivity

Seventy-one participants (49.6%) reported working the same number of hours while WFH as they did in their normal workplace, while 65 participants (45.4%) reported working more hours. The most frequently cited reasons for working more hours while WFH were having more time available due to not having to commute to work and being able to concentrate better due to lack of workplace noise and social interactions and thus becoming absorbed in work and losing track of time.

Sixty percent of the participants reported that they had maintained their typical work schedule (in term of start, finish and break time) while WFH. Thirty-five percent of participants who varied their work schedule mentioned the extra time flexibility allowed by not having to commute. Many reported starting earlier and finishing earlier as a result. Less interruptions at home also allowed participants to work at times that suited them, including outside of normal hours. Participants also mentioned voluntarily varying their schedule in order to fit family/caring responsibilities and self-care activities (e.g. exercise) within break in their work day. Altered break time was frequently mentioned, with many participants reported taking multiple shorter breaks or no breaks while WFH. Some acknowledged this flexibility in break time as positive while others felt that they had been unable to schedule their work properly while at home and had let work ‘overwhelm’ them. Participants described how they “lost track of time” and found it “too easy to keep working” without the routine imposed on them while working on site.

Overall ratings for WFH indicated that participants felt WFH was an improvement on normal working, in particular in relation to their ability to concentrate and be productive (Table 2).

**Table 2:** Aspects on work process and productivity on a Likert scale from 1–10

	**Age group**						
	**21–30** **Mean (SD)**	**31–40** **Mean (SD)**	**41–50** **Mean (SD)**	**51–60** **Mean (SD)**	**61–77** **Mean (SD)**	**Combined** **Mean (SD)**	***P*-value**
Ability to communicate with your manager	8.28 ± (2.05)	8.08 ± (1.90)	8.17 ± (1.92)	7.97 ± (1.65)	8.31 ± (1.70)	8.12 ± (1.84)	0.823
Ability to communicate with your staff	8.85 ± (1.72)	8.48 ± (1.88)	8.25 ± (1.89)	7.62 ± (1.93)	7.50 ± (2.17)	8.19 ± (1.92)	0.238
Ability to collaborate with colleagues	8.50 ± (1.72)	8.28 ± (1.79)	8.00 ± (2.14)	7.63 ± (1.75)	7.62 ± (2.18)	8.02 ± (1.92)	0.321
Ability to keep up to date with your department	8.67 ± (1.75)	7.92 ± (1.77)	7.94 ± (2.25)	7.20 ± (1.65)	7.23 ± (2.71)	7.78 ± (2.01)	0.042
Ability to maintain professional networks	8.22 ± (1.86)	8.05 ± (1.79)	8.11 ± (2.00)	7.76 ± (1.70)	7.54 ± (2.63)	7.93 ± (1.99)	0.716
Access to professional opportunities	8.61 ± (1.42)	8.03 ± (1.80)	7.76 ± (2.42)	7.20 ± (2.16)	7.15 ± (2.94)	7.74 ± (2.22)	0.26
Ability to concentrate on work without distractions	9.28 ± (1.56)	8.92 ± (1.74)	8.86 ± (1.68)	9.23 ± (1.01)	8.62 ± (2.47)	9.01 ± (1.63)	0.668
Ability to get help related to your work	8.78 ± (1.52)	8.03 ± (1.83)	8.06 ± (2.25)	7.70 ± (1.74)	7.92 ± (1.93)	8.03 ± (1.91)	0.257
Ability to get feedback on your work	8.17 ± (1.89)	8.18 ± (1.90)	8.08 ± (2.21)	7.72 ± (1.93)	7.54 ± (2.15)	7.97 ± (2.00)	0.626
Level of autonomy in organizing and completing your work	9.00 ± (1.70)	8.82 ± (1.67)	8.64 ± (1.78)	8.47 ± (1.70)	8.31 ± (2.36)	8.65 ± (1.78)	0.577
Fair allocation of workload	8.22 ± (1.80)	7.64 ± (2.28)	8.03 ± (2.18)	7.76 ± (1.90)	8.31 ± (1.93)	7.89 ± (2.07)	0.765
Work productivity	9.33 ± (1.41)	9.10 ± (1.47)	9.06 ± (1.43)	8.83 ± (1.21)	8.69 ± (1.93)	9.03 ± (1.43)	0.212
Overall job satisfaction	9.33 ± (1.33)	9.03 ± (1.46)	8.57 ± (2.19)	8.70 ± (1.42)	8.31 ± (2.39)	8.81 ± (1.75)	0.203

Statistical significant at level of *P* < 0.05

Eighty-nine percent of participants reported that participating in remote meetings while WFH using various technologies to meet with colleagues remotely, with the most commonly used technology being videoconferencing services such as Skype for Business, Pexip, Zoom and MS Teams (Table 3).

**Table 3:** Most commonly used technology to participate in remote meeting

Technology type	# of participant who reported using
1. Telephone	108
2. Videoconference	120
3. Instant messaging/chat	87

Participants reported a perceived improvement in particular to the time-efficiency of meeting remotely in comparison to their experiences of face to face meetings (Table 4).

**Table 4:** Aspects on remote meeting

	**Age group**						
	**21–30** **Mean ± SD**	**31–40** **Mean (SD)**	**41–50** **Mean (SD)**	**51–60** **Mean (SD)**	**61–77** **Mean (SD)**	**Combined** **Mean (SD)**	***P*-value**
Ability to participate in meetings	8.538 ± 1.664	8.216 ± 1.797	8.000 ± 2.142	7.741 ± 1.789	8.636 ± 0.924	8.096 ± 1.833	0.682
Ability to communicate with other meeting participants	8.69 ± 1.25	8.03 ± 1.94	8.14 ± 2.02	7.68 ± 2.04	8.55 ± 1.13	8.08 ± 1.87	0.729
Time efficiency of meetings	9.000 ± 0.816	8.676 ± 1.634	8.314 ± 2.097	8.038 ± 1.612	8.273 ± 1.421	8.427 ± 1.688	0.331
Team interpersonal engagement	8.31 ± 1.70	7.54 ± 2.18	7.72 ± 2.33	7.11 ± 2.10	7.73 ± 2.20	7.56 ± 2.18	0.558
Effectiveness of meeting interaction and outcomes	8.08 ± 1.78	8.06 ± 2.00	8.09 ± 2.04	7.48 ± 1.85	7.73 ± 2.28	7.88 ± 1.98	0.629

Statistical significant at level of *P* < 0.05

Over 55% of participants (*n* = 78) indicated that they had learned new work-related skills as a results of WFH such as improved proficiency with videoconferencing applications. Other technology-related skills were also frequently mentioned, particularly the ability to troubleshoot computer equipment and systems. Many participants also felt they improved their general ways of working while WFH, including their communication skills, time management and ability to work autonomously.

### Impact of teleworking on family and home life

While 53% of participants (*n* = 75) reported having children living in their home, only 17% reported providing care or supervision for them while WFH. Fifty-four percent of participants reported having at least one other person working or studying in their home.

**Fig. 3:**
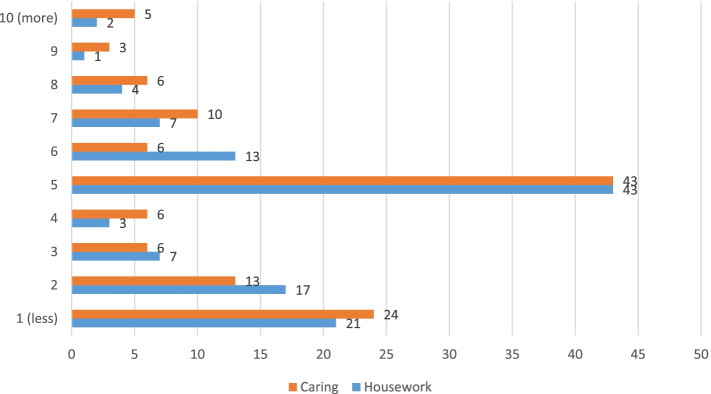
Perceived change in amount of time devoted to home activities while WFH

Participants reported that no perceived changes with their time they spent on housework and caring activities when teleworking from home comparing to when they worked on site (Fig. 3). However, a significant number of participants felt they were spending considerably less time on these activities while WFH.

### Impact of teleworking on personal wellbeing

The SPANE relating to affect balance while WFH was completed by 124 participants (85.7%), resulting in a mean score of 5.45 (S.D. 2.98). The SPANE relating to normal working conditions was completed by 127 participant (88.8%) resulting in a mean score of 2.70 (S.D 3.69). The results showed that while participants’ positive emotions typically predominated in both situations, they felt slightly more positive with WFH. Participants perceived their personal wellbeing had improved while WFH with particularly positive response in relation to physical comfort while working and the ability to get exercise (Table 5). Over 90% participants reported that they would take the opportunity to WFH again if it were offered.

**Table 5:** Aspects on personal well being

	**Age group**						
	**21–30** **Mean ± (SD)**	**31–40** **Mean (SD)**	**41–50** **Mean (SD)**	**51–60** **Mean (SD)**	**61–77** **Mean (SD)**	**Combined** **Mean (SD)**	***P*-value**
How physically comfortable was the work space	8.53 ± (1.41)	8.64 ± (1.94)	8.03 ± (2.25)	7.87 ± (2.21)	8.42 ± (1.16)	8.27 ± (1.98)	0.682
Ability to exercise regularly	8.86 ± (1.66)	8.25 ± (1.98)	8.74 ± (1.67)	7.67 ± (2.73)	7.85 ± (1.82)	8.29 ± (2.07)	0.228
Ability to spend time on hobbies and leisure activities	8.13 ± (1.77)	7.91 ± (2.03)	7.74 ± (2.32)	6.38 ± (2.87)	5.77 ± (2.42)	7.32 ± (2.44)	0.015
Ability to spend time resting and relaxing	8.20 ± (1.90)	8.08 ± (1.93)	8.00 ± (2.01)	7.00 ± (2.78)	7.83 ± (1.64)	7.79 ± (2.16)	0.486
Social connection to others	7.53 ± (2.23)	7.57 ± (2.21)	7.63 ± (2.12)	6.55 ± (2.67)	6.54 ± (2.40)	7.22 ± (2.33)	0.328

Statistical significant at level of *P* < 0.05

### Thematic analysis

Data obtained from the open-ended questions had complimented the findings of the structure close-ended questions in the benefits of remote working and support for their health and wellbeing. The open-ended questions had provided additional information on challenges which the participants encountered during the WFH experience and their suggested future preference to sustain this workplace practice.

### Benefits of remote working

Working from home was perceived beneficial by most of the participants. They felt more productive while WFH as they did not have to travel, resulting in less disruptions. They could direct travel hours into productive hours or used it for other activities instead. Participants also learned new work-related skills mainly videoconferencing applications and ability to troubleshoot computer equipment. They also enhanced their communication and time management skills, and acquired ability to work autonomously.

*“Didn’t have to waste time travelling so made use of that time working more. Sometimes it’s easy to keep going before you realise you should stop. So many less interruptions helps you focus on your task”. (Participant 10)*

Participants experienced less distraction at home and they could accomplish their task without interruptions as there were no people coming around for chats like in office which could be very noisy at times.

*“The best part of working from home is that I have got quality time to concentrate on my work, as the office sometimes can be very noisy and people keep talking with each other and over phones”. (Participant 2)*

Many participants reported that flexibility of time also impacted their wellbeing and performance as they could decide when and how to accomplish their task, some started earlier or worked after hours as needed.

*“Due to the flexible hours it was easier for me to work an extra hour or so at night to answer emails etc.”. (Participant 23)*

Digital communication seemed effective and easier as there was increased information sharing across the team, the team members were approachable and easily available via phone or email. People were more punctual/time efficient in digital meetings as a result there was possibility of accommodating more meetings in a day. There were no issues related to finding a room or location for a meeting or travel to the designated location.

*“In general, everyone being on line meant that you could squeeze more meetings in back to back not having to worry about finding a room, locations etc. The whole electronic collaboration is a lot more effective and efficient than a traditional meeting space”. (Participant 121)*

Working from home was also reported to have financially impacting the participants as they felt that they could save the money spent on fuel, parking, car maintenance, tickets and opal cards, buying coffee or food.

*“Working from home helped with my finances as well as I am spending less money on fuel/travel, money, coffee, etc.…” (Participant 40)*

### Support health and well being

One of the major theme was the impact of WFH on the mental and physical wellbeing of the participants. They could sleep for longer hours without stress to spend hours in traffic and look for the parking.

*“No travelling time means sleep in, less stress, more focused when working and more time at home after work so days don't feel so rushed and stressful. I find I'm much more productive when I work from home as there are less interruptions”. (Participant 67)*

The participants also stated that WFH enabled them to build a healthier work life balance. They were more socially and physically active as more time for exercise, walk their dogs, cook fresh meals or spend time with their family.

*“I was more productive while working from home as there are fewer destruction. Also saving the travel time and utilise that with my family. My work life balance get much better”. (Participant 88)*

*“Instead of spending time commuting I was using that time to exercise & walk the dogs”. (Participant 98)*

### Challenges

Some of the participants found it unsettling as it was hard for them to keep WFH separate. They had to simultaneously care/support their kids or family in the house due to COVID-19 related school closures. However they reported that caring for family did not impact their work or productivity.

*“I needed to assist children with online learning which took some time away from work”. (Participant 102)*

*“Tried to but difficult to manage a 3 year old between my wife's and my meetings. The challenge for us became to try and schedule meetings not at the same time so one of us could entertain the wee little one”. (Participant 140)*

Mostly of the participants felt that they worked for longer hours as compared to usual as it was harder to keep track of time. They also reported being overloaded with work sometimes.

*“Workload was very high. Since the laptop was setup and easily accessible at home, tended to start work earlier than normal (no travel time) and continued working sometimes till later in the evening”. (Participant 77)*

Communication & engagement was difficult in virtual meetings especially with large groups. It sometimes became difficult for the people to participate as too many conversations happened at the same time or some people would hijack the conversation and others felt left out. Occasionally there were also interruptions due to the surrounding environment. There were issues related to trust, work allocation and accountability.

*“Videoconference and teleconference meetings difficult at times to participate in as each other cannot read each other nuances meaning that people at times spoke over the top of each other. Also at times meetings interrupted with people's barking dogs, neighbourhood sounds, and just general distractions which you don't get when in workplace”. (Participant 111)*

*“I found that some of the team became disgruntled during the period. This was mainly related to presuming others in the team weren't pulling their weight when the 'visibility' of others working was lost. There was some truth in it. Working remotely is not suited to all staff - work ethic and attributes play a large role in the suitability of staff”. (Participant 96)*

The lack of trust and expectation from the manager/team leader was also identified as a challenges for some people. The higher expectations made them anxious and they felt that being monitored hourly and completing a very in-depth spreadsheet was time taking and unpleasant.

*“I had high anxiety because the team leader would call very often and have much higher expectations of us who were working from home. Also, we had to complete a very in depth spreadsheet detailing what we did every 15minutes, which was too time consuming.” (Participant 5)*

*“I was monitored so thoroughly every hour and also if the spreadsheet we have to fill out and send in each day was less detailed”. (Participant 100)*

Some of the participants experienced difficulties with getting onto the network drives sometimes bandwidth issues of home internet not ICT, along with access to shared drive and printers.

*“No access to shared drives as a result of not having a work laptop made it challenging to access files at home to work on”. (Participant 21)*

Few participants felt lonely and missed all the social interactions happening in the work place and capacity to make new connections. It also impacted on the opportunities to seek help and support from the team along with the opportunities for feedback, learning & performance management.

*“I felt lonely not being with my work colleagues”. (Participant 22)*

*“In an office environment there is a considerable amount of skill tips from the team, which is lost WFH.” (Participant 109)*

### Future preferences

The open-ended questions had provided additional information on challenges described in the above paragraphs. The participants expressed strong desire for WFH to be allowed in future as it created a better work life balance. They suggested incorporating flexible work options including telework as part of annual performance development review and recruitment strategy as it would attract more skilled staff to work for the health service. In order to implement it appropriately there is a need for commitment, planning and processes in place for worker accountability. More support and trust from the managers and greater IT literacy is required for WFH to succeed.

## Discussion

In this study, there was overwhelmingly positive experience of the staff realising the benefits of WFH despite of some challenges. They felt more productive due to less interruptions and flexibility of time. Team members were approachable and easily available. People learned new work-related skills, enhanced their communication and time management skills, and acquired ability to work autonomously. WFH had a good impact on their mental and physical wellbeing and enabled them to build a healthier work life balance. These results mirror the study conducted by the University of New South Wales on 6,000 Australian Public Service employee [21], that staff felt their productivity and autonomy was increased and they enjoyed the person benefits including less commuting time, more time with family and for caring responsibilities.

Staff WFH during the pandemic could not be considered to have a typical experience of WFH as a flexible work practice, in that they were not doing so voluntarily and a variety of other stressors might be in place which could influence their experience of WFH. However not all benefits or drawbacks experienced by staff WFH would be specific to the pandemic situation, and the transition of a large number of staff to WFH at once presented an unique opportunity for investigation and data collection.

Detailed examination of the impact of implementing WFH during the early stages of the pandemic as per this study, had the potential both to inform policymaking and to provide baseline data for ongoing evaluation of the health service’s approach to WFH and flexible work practice as those policies evolve. It is also possible that remote working on masses may be necessary again in future during pandemic or other disasters. During the 2019–20 bushfires, for example, WFH was identified as a way of avoiding worker exposure to bushfire related hazards [19]. In similar future scenarios insights gathered from the COVID-19 experience may be particularly useful.

The representation of the participants in this study might impose a limitation of the study because broader representation would allow more reliable generalisability of the results and minimise election bias towards those WFH, resulting in an under-representation of those who did not WFH.

This study was intended to gather feedback from the corporate and non–clinical staff, thus the resulting insights might not be applicable to all health care workers in particular staff working in frontline, operational and clinical roles. This study also focused on the experience from the staff but not their managers. Understanding the mindset of their managers on the benefits of WFH and their receptiveness towards it in the future would be beneficial. Future direct exploration of in-depth perspectives of their experience would also be vital.

The findings from this study were being used to formulate the evaluation of the interim policy and framework on flexible working practice in particular WFH. Exploration on the relationship between WFH and mental and physical wellbeing could be a research priority to sustain a healthy workforce and scope for innovative workplace co-design.

## Conclusion

This study highlighted factors that impacted workers’ work processes, productivity, physical and mental health well-being while WFH and provided a foundation for considering how to best support a positive WFH experience. The participants expressed strong desire for WFH to be allowed in the future. For it likely to be successful in future, organisations will need to implement formalised WFH policies that consider work-home boundary management support, role clarity, workload, performance indicators, technical support, facilitation of co-worker networking, and training for managers.

## Data Availability

The datasets used and/or analysed during the current study are available from the corresponding author on reasonable request.
